# Aging and post-polymerization effects on conversion degree and properties of additive splint materials

**DOI:** 10.1590/1807-3107bor-2025.vol39.017

**Published:** 2025-02-07

**Authors:** Leandro Ruivo de SANTIS, Lucas Silveira FERNANDES, Mayra Torres VASQUES, Nataly Rabelo Mina ZAMBRANA, Ítallo Emídio Lira VIANA, Taís Scaramucci FORLIN, Guilherme de Siqueira Ferreira Anzaloni SAAVEDRA, Carlos Eduardo FRANCCI

**Affiliations:** (a) Universidade de São Paulo – USP, School of Dentistry, Department of Prosthetics, São Paulo, SP, Brazil.; (b) Private practice, São Paulo, SP, Brazil.; (c) University of Michigan, School of Dentistry, Biologic and Materials Science and Prosthodontics Department, Ann Argor, MI, USA.; (d) Tufts University, School of Dental Medicine, Medford, MA, USA.; (e) Universidade de São Paulo – USP, School of Dentistry, Department of Dentistry, São Paulo, SP, Brazil.; (f) Universidade Estadual Paulista – Unesp, School of Dentistry, Department of Dental Materials and Prosthetics, São Paulo, SP, Brazil.; (g) Universidade de São Paulo – USP, School of Dentistry, Department of Biomaterials and Oral Biology, São Paulo, SP, Brazil.

**Keywords:** Occlusal Splints, Printing, Three-Dimensional, Polymerization

## Abstract

The study objective was to analyze dimensional change, flexural strength, surface hardness, wear profile, and conversion degree of different additive splint materials under various post-polymerization conditions of time and artificial aging. Two additive manufacturing systems (Cara Print 4.0, Dima Print Ortho, Kulzer; SprintRay Pro, SprintRay Splint, SprintRay), and a thermally activated resin control (Clássico) were evaluated in artificial aging (deionized water or saliva; 28 or 84 days at 37°C), with recommended or doubled post-polymerization cycles. Dimensional change (surface metrology), flexural strength (ISO 20795-1:2013), fractography (SEM), Knoop hardness, two-body wear profilometry (150,000 cycles; 3mmØ; 20N; 2.1Hz), and conversion degree (FTIR spectroscopy) were assessed. Two-way ANOVA and post-hoc Tukey tests were used for parametric data, and Kruskal-Wallis and post-hoc Dunn tests, for non-parametric data (α = 0.05). Results indicated no statistically significant differences in dimensional change or flexural strength among the materials. Recommended post-polymerization cycles resulted in lower hardness for additive resins than the thermally activated control. Doubling post-polymerization time significantly increased flexural strength and hardness of Dima Print Ortho, but decreased flexural strength of SprintRay Splint, and did not affect wear resistance. Dima Print Ortho demonstrated the highest wear resistance. Artificial aging did not affect flexural strength, surface wear, or dimensional change, but negatively impacted the hardness of all materials except Dima Print Ortho. The conversion degree was unaffected by post-polymerization time, and no significant differences were found among the materials. Overall, additive materials exhibited mechanical and dimensional properties comparable to thermally activated resin, with doubling post-polymerization time positively influencing the properties.

## Introduction

Temporomandibular disorders can often reduce quality of life by causing pathological changes in dental, bone, and neuromuscular structures. In this context, rigid occlusal splints may help preserve the tooth structure from parafunctional wear; therefore, splint materials must be resistant to wear.^1-9^CAD/CAM technology^10^ has promoted dynamic advancements in both conventional clinical and laboratory processes, enhancing precision and efficiency in the production of thermally activated resin occlusal splints.^11-16^ Stereolithography and digital light processing (DLP) use additive manufacturing, which involves liquid photoreactive resin, a model-building platform, and ultraviolet (UV) light. In this process, a tank containing photoreactive liquid resin is selectively exposed to ultraviolet (UV) light, causing the resin to polymerize in layers^17^ that build up to form a solid object.^18,19^ Additional post-processing procedures, including cleaning and post-curing, are essential.^20,21^ Post-curing involves further exposure to light after the initial construction of the solid, thus ensuring full polymerization of the material.^18,19^ This additional step can influence various mechanical properties and the final dimensions of the constructed polymeric material.^20,22-24^

In addition, occlusal splints may fracture due to accidents or fatigue. Among the primary mechanical properties that pose challenges in an intraoral environment are flexural resistance, which considers the concentration of tensions that lead up to the complete rupture of the material;^3,7,24-30^ superficial hardness, related to rigidity and material resistance,^7,23-25,28,29,31^ and resistance to degradation when exposed to salivary humidity. Degradation in these situations is largely attributed to water sorption by polymers.^23,24,27,29,30,32^ Furthermore, the selected technology and splint material must ensure passive adaptation and dimensional stability,^21,22,33,34^ considering that surface dimensional metrology is an effective method for quantifying precision and dimensional accuracy. This method consists of digitally measuring the differences between two surfaces of polygonal meshes.^34,35^

The conversion degree (CD) is the degree of polymerization, and significantly impacts the mechanical properties. The strength and surface hardness of photopolymers can be enhanced by increasing the conversion of monomers.^22,24,25,36-38^ Furthermore, polymers with a high conversion degree tend to undergo reduced degradation through water sorption, because fewer unreacted monomers are leached out.^39^Considering the importance of investigating additive materials during clinical challenges, this study aimed to evaluate the influence of two printing technologies on flexural strength, surface hardness, wear profile, and dimensional change of different polymers for additive manufacturing, according to post-curing time and artificial aging, as compared with thermally activated resin used to produce occlusal splints. The null hypothesis posited that different materials, post-curing periods, and artificial aging do not influence the properties evaluated.

## Methods

Dimensional change, flexural strength, fractography, Knoop hardness, wear profile, and conversion degree were evaluated in the different additive splint materials, and the thermally activated acrylic resin as the control group for rigid occlusal splints (Table 1). This evaluation considered the influence of two different additive manufacturing systems, post-curing time points (recommended and doubled), and artificial aging in deionized water for 28 days to test bending and dimensional change, or artificial saliva for 28 and 84 days to test hardness, or else only 84 days for surface wear pattern.

**Table 1:** Type, name, manufacturer, group, artificial aging time, and post-curing of tested occlusal splint materials.

Type	Name and Composition	Manufacturer	Group	Aging	Post-curing
Additive manufacture	Dima Print Ortho	Kulzer; Wehrheim, Germany	K0R	-	Recommended
			K28R	28 days	
	Esterification products of 4,4-isopropylidenediphenol, ethoxylated 2-methylprop-2-enoic acid, methacrylic oligomers, and phosphine oxides.		K84R	84 days	
			K0D	-	Doubled
			K28D	28 days	
			K84D	84 days	
	SprintRay Splint	SprintRay – Los Angeles, USA	S0R	-	Recommended
			S28R	28 days	
	Methyl methacrylate oligomers, and photo initiators.		S84R	84 days	
			S0D	-	Doubled
			S28D	28 days	
			S84D	84 days	
Thermically activated	RAAT	Classic Artigos Odontológicos	TA	-	Absent
			T28A	28 days	
	Powder: pre-polymerized spheres of methyl methacrylate and benzoyl peroxide (initiator). Liquid: unpolymerized methyl methacrylate, and hydroquinone (inhibitor).		T84A	84 days	

Specimens measuring 65x10x3.3 mm (± 0.2 mm) were made in compliance with ISO 20795-1:2013 to evaluate dimensional change and flexural strength.^40^ Each group consisted of 10 specimens (n = 10), resized from initial measurements, with a 2 mm excess in all dimensions. Resizing was achieved by finishing and polishing with SiC abrasive sandpaper in a polishing machine (Buehler Automet 250; Buehler) with grit sizes P400, P600, P800, P1000 and P1200 for 2.5 minutes under running water. Then the specimens were placed in an ultrasonic tank (L100; Schuster) with deionized water for 180 seconds to remove residues. Using additive manufacturing techniques, eighty specimens were made from a virtual model (Tinkercad – Autodesk) on two DLP technology printers, and their respective resins were used for the rigid occlusal splints (Cara Print and Dima Print Ortho – Kulzer, and SprintRay Pro and SprintRay Splint – SprintRay). Forty specimens were made for each printer, following the manufacturer’s recommendations of a 50 μm layer thickness at 45° relative to the printing platform.

After printing, all specimens were immersed in two sequential ultrasonic baths with isopropyl alcohol (> 96%) for 3 and 2 minutes, respectively, to remove excess unpolymerized resin. Subsequently, they were immersed in another ultrasonic bath with isopropyl alcohol (> 96%). After drying, they underwent a post-curing process in a UV chamber, following the tested regimens: one was recommended by the manufacturer (10 minutes; 200 W; 390–540 nm – HiLite Power 3D Kulzer or 14 minutes; 90 W; 365–405 nm – ProCure SprintRay), and the other was doubled (20 minutes – Kulzer or 28 minutes – SprintRay). Twenty specimens of thermally activated acrylic resin (Clássico Dental Articles) were fabricated using condensation silicone molds (Zetalabor; Zhermack) made from metal patterns in stainless steel (67 mm x 12.0 mm x 5.3 mm), incorporated into a screwed metal muffle (OGP) with type II plaster (Asfer Chemical Industry). The molds were filled with thermally activated polymerized acrylic resin, prepared according to the manufacturer’s instructions, and cured in a water bath heated to 72°C for 9 h^37,39^ using an electric thermopolymerizer (Ter- motron, P-100; Termotron Equipamentos, Piracicaba, Brazil). The specimens were then subjected to the same finishing and polishing procedures as described earlier. Prior to testing, each group was stored in an oven (deionized water; 37°C; 48 ± 2 h), with half of the specimens undergoing artificial aging for 28 days under the same conditions.

The dimensional change was measured by using surface dimensional metrology, facilitated by 3D measurement data analysis software (Gom GmbH). Each specimen (n = 10) was scanned with a desktop scanner (S300 Arti – Zirkonzahn) to create a .STL file. The digitized specimens were automatically aligned to the original .STL digital model using the best alignment algorithm. Specifically, the analysis was conducted using a chromatic spectrum with maximum/minimum critical values programmed to ± 500 μm, and maximum/minimum nominal values set at ± 10 μm. The data for analysis were displayed on a color map overlaid on the mesh of the specimen, representing standard deviations of the means of both positive and negative values. The influence of various additive technologies and post-curing regimes was analyzed by identifying regions of greater or lesser volumetric discrepancy, and calculating the percentage linear dimensional change along the two primary axes (width and height), for both non-aged and artificially aged specimens.

A 3-point bending test was conducted using a universal testing machine (Instron 5565; Instron) with a centered load, at a crosshead speed of 5 mm/min and a span length of 50 mm. Specimens were immersed in deionized water at 37°C, and subjected to a strain interval of 0.5 to 1.0 mm. The maximum flexural strength (σf) was calculated in megapascals (MPa), based on the maximum central flexural load (N) applied to the specimen at the point of rupture, as indicated on the displacement-load curve. The flexural strength was determined using the equation: $\left[\mathrm{of}=3\mathrm{PL}/2{\mathrm{wh}}^{2}\right]$ where “P” represents the maximum bending load (N), “L” is the span length of the support (50 mm), “w” is the specimen width (10 mm), and “h” is the specimen height (3.3 mm). Fractography was assessed using scanning electron microscopy (SEM) (Zeiss Sigma, Zeiss). The fracture interface of a randomly selected fragment from each material (S0R, K0R, and T0A) was analyzed qualitatively.

Regular prisms with a square base measuring 100 mm^2^ and a height of 3.3 mm^2,5-7^ (n = 10) were prepared for Knoop hardness (HK) and wear profile assessments from the fragments obtained during the flexural test.^25,26^ Each prism was embedded in chemically activated acrylic resin (Clássico, SP, Brazil), with the wear surface oriented perpendicular to the light of the PVC support, which had a diameter of ¾ inches and a height of 24 mm. The exposed surfaces of the fragments were polished with silicon carbide sandpaper with grit sizes of 120, 320, 800, 1,000, 2,000 and 4,000, as previously described. Hardness measurements were performed with a hardness tester (HMV-2000; Shimadzu Corporation, Kyoto, Japan), applying a static vertical load of 25 grams for 5 seconds. Ten indentations per specimen were executed, five of which were made in each fragmented half, ensuring a distance of 500 μm between each evaluated region. The Knoop hardness value was determined for each indentation. The software automatically calculated the measurement of the larger diagonal, displaying the Knoop hardness value on the computer monitor in real-time. The mathematical equation used for the calculation was as follows: $\left[\mathrm{HK}=P/A=P/\left(\mathrm{CpL}C^{\wedge }2\right)\right]$ where “P” represents the applied load (kgF), “A” is the surface area of the indentation (mm^2^), “ L” is the length of the indentation (mm) along the longest axis, and “Cp” is the correction factor (0.070279).

Prior to masticatory test simulation, initial readings were obtained using an optical profilometer (Proscan 2100; Scantron, Tauton, England) to analyze the surfaces intended for wear according to their initial profile, thereby standardizing the initial curvatures to below 0.5 μm. The two-body tribological test was conducted on a masticatory simulation machine (CS-4.8, SD Mechatronik, Feldkirchen-Westerham, Germany), using stainless steel spherical indenters (6 mm Ø; 20 N; 150,000 cycles; circular movement of 3 mm Ø), with a potential penetration depth of 1 mm, immersed in water at 37°C. Following the test, the worn surfaces of each specimen were analyzed with the optical profilometer to assess surface loss. The device sensor covered both the wear area and the reference surfaces on either side. The profiling was programmed to execute 500 steps, with a size of 0.01 mm on the x-axis, and 60 steps with a size of 0.1 mm on the y-axis. The depth of the lesion was calculated by subtracting the average height of the test area from the average height of the reference surfaces using specific software (Proscan Application software version 2.0, 17; Scantron, Tauton, England).

Three specimens from each group of resins were selected to represent the polymerized specimens. The unpolymerized resin, corresponding to a drop of the printed material, was used to measure the spectra of the specimen prior to polymerization, while the thermally activated acrylic resin was measured immediately after mixing. The conversion degree was measured using Fourier transform infrared spectroscopy in total attenuated reflectance mode (FTIR-ATR; Vertex 70, Bruker Optics, Germany), with a resolution of 4 cm^-1^ and 16 scans, employing the absorbance method.

The thermally activated acrylic resin and the SprintRay resin were analyzed based on the peaks at 1720 cm^-1^ and 1638 cm^-1^, which correspond to the C=O and C=C bonds, respectively. Consequently, the conversion degree for these resins was determined using the following formula:^37^


$$\text{ Conversion degree }(\%)=100\left[1-\frac{\left(\frac{\text{ A1683 }}{\text{ A1720 }}\right)\text{ polymerized }}{\left(\frac{\text{ A1683 }}{\text{ A1720 }}\right)\text{ unpolymerized }}\right]$$

Dima Print resin was analyzed based on the peaks at 1,610 cm^-1^ and 1,638 cm^-1^, which are associated with aromatic and aliphatic C=C bonds, respectively, as determined by the following formula:^39^


$$\text{ Conversion degree }(\%)=100\left[1-\frac{\left(\frac{\text{ A1683 }}{\text{ A1720 }}\right)\text{ polymerized }}{\left(\frac{\text{ A1683 }}{\text{ A1720 }}\right)\text{ unpolymerized }}\right]$$

Two-way ANOVA and post-hoc Tukey tests were used for parametric data, while Kruskal-Wallis and post-hoc Dunn tests were employed for non-parametric data (α = 0.05).

## Results

In the analysis of dimensional change, the groups exhibited adherence to the normal distribution (p = 0.568) and demonstrated heteroscedasticity (p = 0.03). Table 2 presents the median values, interquartile deviations, and the result of the Kruskal-Wallis test for dimensional change following artificial aging and/or post-curing of the various materials. Dimensional changes resulting from doubled post-curing and artificial aging were not statistically significant.

**Table 2:** Medians and interquartile deviations of dimensional change and Dunn test results (5%).

Material and post-curing	Artificial aging	
	Absent	Water 28 days (28)
	mm	mm
K0R	Absent	-0.0125 ± 0.0275 ^(A)^
K0D	-0.0270 ± 0.056 ^(A) (a)^	-0.0085 ± 0.0183 ^(A) (a)^
S0R	Absent	-0.01 ± 0.046 ^(A)^
S0D	-0.0475 ± 0.0368 ^(A) (a)^	-0.0435 ± 0.0285 ^(A) (a)^
TA	Absent	-0.0105 ± 0.017 ^(A)^

Uppercase letters refer to columns; lowercase letters refer to rows; different letters show statistically significant differences (p < 0.05).

In the flexural strength assessment, all groups demonstrated adherence to the normal distribution (p = 0.777) and homoscedasticity (p = 0.202). Table 3 presents the mean values (MPa) and standard deviations obtained for flexural strength, along with the results of the Tukey multiple comparison test across the different groups. According to the 2-way ANOVA and post-hoc Tukey analysis, no statistically significant differences were observed among the materials, whether without aging or after doubled curing. Additionally, no statistically significant aging-related differences were noted within each material. However, significant differences were found between groups S0D and K0D (p < 0.001), between K28R and K28D (p < 0.04), and between K28D and T28A (p < 0.001).

**Table 3:** Means and standard deviations of flexural strength (MPa) and Tukey test results (5%).

Material and post-curing	Artificial aging in water (37°C)	
	Absent (0)	28 days (28)
	MPa	MPa
KR	82.80 ± 11.13 ^(A,B) (a)^	76.34 ± 8.54 ^(A, C) (a)^
KD	93.05 ± 15.91 ^(B) (a)^	93.9 ± 10.33 ^(B) (a)^
SR	81.36 ± 15.75 ^(A,B) (a)^	78.15 ± 7.71 ^(A,B,C) (a)^
SD	69.54 ± 12.52 ^(A) (a)^	82.68 ± 12.92 ^(A,B,C) (a)^
TA	78.01 ± 12.03 ^(A,B) (a)^	67.76 ± 8.06 ^(C) (a)^

Uppercase letters refer to columns; lowercase letters refer to rows; different letters show statistically significant differences (p < 0.05).

In the Knoop hardness test, all groups exhibited adherence to the normal distribution (p = 0.294), but did not demonstrate homoscedasticity (p < 0.001). Table 4 presents the median values and interquartile deviations for the surface Knoop hardness, along with the results of Dunn’s multiple comparison test for the different groups. Analysis using Kruskal-Wallis and Dunn post-hoc tests revealed that, without aging or doubled post-curing, the conventional acrylic resin (TG) exhibited a statistically significant difference from the other additive materials (p < 0.001). Excluding the K0D group, all additive groups showed a statistically significant difference from the control group (TG). When comparing the aged groups, the different materials, and the post-curing time points, aging resulted in a statistically significant reduction in hardness, with the lowest values observed in groups aged in artificial saliva for 84 days.

**Table 4:** Medians and interquartile deviations of Knoop microhardness and Dunn test results (5%).

Material and post-curing	Artificial aging (37°C)		
	Absent	Water	Saliva
		28 days (28)	84 days (84)
	HK	HK	HK
KR	20.48 ± 0.27 ^(A) (a)^	21.05 ± 0.61 ^(A) (a)^	18 ± 0.27 ^(A) (a)^
KD	21.08 ± 0.16 ^(A,B) (a)^	21.24 ± 0.36 ^(A) (a)^	17.98 ± 0.21 ^(A) (b)^
SR	20.5 ± 0.5 ^(A) (a,b)^	21.51 ± 0.34 ^(A) (a)^	17.7 ± 0.21 ^(A) (b)^
SD	20.11 ± 0.2 ^(A) (a,b)^	21.15 ± 0.4 ^(A) (a)^	17.62 ± 0.16 ^(A) (b)^
TA	28.27 ± 1.2 ^(B) (a)^	21.9 ± 0.45 ^(A) (a)^	16.72 ± 0.39 ^(A) (b)^

Uppercase letters refer to columns; lowercase letters refer to rows; different letters show statistically significant differences (p < 0.05).

**Figure 1:**
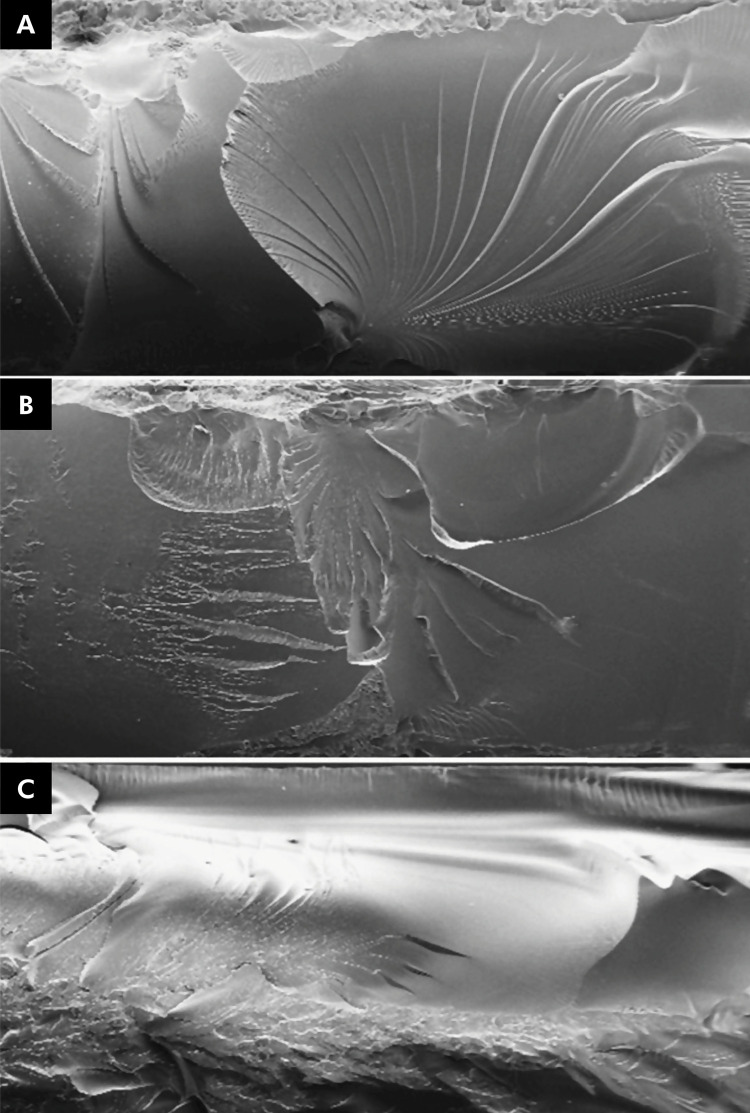
Scanning electron microscopy (SEM) of fracture interfaces. A) Dima Print Ortho; B) SprintRay Splint; C) Thermally activated resin.

In the evaluation of surface wear profiles, the groups demonstrated adherence to the normal distribution (p = 0.568) and exhibited heteroscedasticity (p = 0.03). Table 5 presents the median values, interquartile deviations, and results from the Kruskal-Wallis test, along with Dunn’s multiple comparisons for surface loss after the wear test of the various materials. The thermally activated acrylic resin exhibited the greatest surface loss, showing a statistically significant difference only when compared with the K0R group. Doubling the post-curing time did not significantly influence surface loss due to wear in any of the additive groups. No groups displayed statistically significant differences related to artificial aging within the same material. Microscopic magnification of the fractography revealed differences in the fracture interfaces of the evaluated materials. The additive materials demonstrated stress concentration between the additive layers, whereas the conventional acrylic material exhibited stress concentration within porosities incorporated into its structure (Figure 1).

**Table 5:** Medians and interquartile deviations of surface loss and Dunn test results (5%).

Material and post-curing	Artificial saliva aging (37°C)	
	Absent	84 days (84)
	µm	µm
KR	349.78 ± 26.6 ^(B) (a)^	338.41 ± 51.05 ^(A) (a)^
KD	390.42 ± 118.61 ^(A,B) (a)^	342.33 ± 21.03 ^(A) (a)^
SR	449.77 ± 39.34 ^(A) (a)^	391.41 ±1 8.58 ^(A,B) (a)^
SD	440.76 ± 29.64 ^(A) (a)^	378.47 ± 48.35 ^(A,B) (a)^
TA	517.11 ± 50.61 ^(A,C) (a)^	437 ±8 6.9 ^(B) (a)^

Uppercase letters refer to columns; lowercase letters refer to rows; different letters show statistically significant differences (p < 0.05).

**Figure 2:**
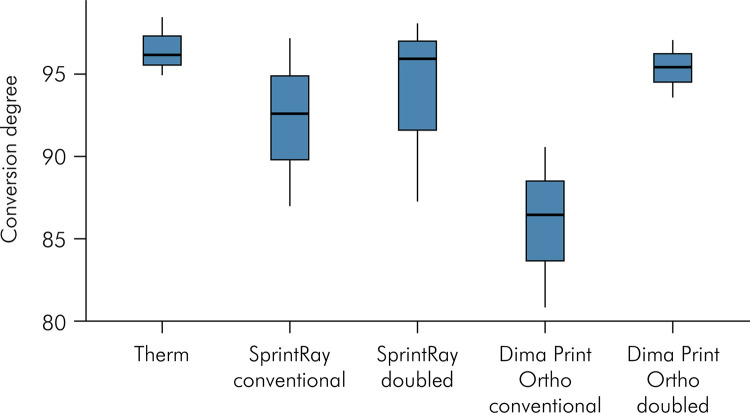
Conversion degree and median values for each resin material.

In the assessment of conversion degree, the descriptive analysis indicated parametric data, showing adherence to the normal distribution and homogeneity of variances. One-way ANOVA inferential statistical analysis revealed that the degree of resin conversion was not significantly influenced by post-cure time (p = 0.189) (Figure 2).

## Discussion

The present study aimed to evaluate the influence of two digital light processing stereolithographic additive manufacturing technologies, two post-curing time points, and artificial aging on the dimensional change, flexural strength, surface hardness, wear profile, and conversion degree of various polymers for additive manufacturing. The evaluation of these polymers was compared with that of the thermally activated acrylic resin used in fabricating occlusal splints, along with complementary qualitative fractography analyses.

The authors sought to simulate the intraoral environment^7,24,26,28,29^ by replicating approximately 3 months of intraoral use ^27^. Printing at 45° may help balance resistance to axial loads, while minimizing dimensional change ^22^ and reducing water sorption.^27^ In layering construction, smaller thicknesses (50 μm) can optimize flexural strength,^6,22^ since larger thicknesses may decrease the intensity of light penetrating the material, thus altering the absorption and diffraction of curing light.^19,24^ According to the manufacturer’s orientations, the flexural strength values of all the materials were similar, corroborating the findings from one study ^27^ while contradicting others.^7,28,29^ Nonetheless, all the materials met the specifications of ISO-20795-1:2013,^40^ (≥ 65 MPa), which is both consistent with^27-29^ and contrary to the literature.^7^These discrepancies may be attributed to the diverse range of materials tested across studies, despite employing similar methodologies.

Reduction in flexural strength after aging can be attributed to the incorporation of water during the process, since its molecules penetrate the polymer structure and act as plasticizers, leading to a decrease in this mechanical property.^23,24,27,30,32^ However, this phenomenon was not observed in this study, which partially supports the null hypothesis, contrary to findings from previous studies.^25,27,29^ Doubling the post-curing time significantly affected the flexural strength of the additive materials, partially rejecting the null hypothesis, with an increase in the K0D group and a decrease in the S0D group. This variation may be attributed to the different compositions and polymerization strategies of each material.^19,35^ In contrast, the additive groups exposed to double post-curing and aging showed a non-significant increase in flexural strength, which was not consistent with the results observed for the conventional material. The doubled post-curing may signal a change in the polymeric network of the aged materials, potentially enhancing the tolerance of the additive materials against water sorption.^29^

Regarding the composition of the printed resins, different peaks were observed during the conversion degree test for the S and K materials. The S resin did not exhibit a peak at the 1610 cm^-1^ band, which corresponds to aromatic C=C bonds. This absence suggests that the S resin may lack aromatic chain monomers in its composition. This assertion is supported by the manufacturer’s specification of oligomers of methyl methacrylate on the product label, which indicates a linear structure. In contrast, the K resin displayed a peak at 1610 cm^-1^, indicating the presence of aromatic chain monomers. The manufacturer’s package insert mentions products of 4,4-isopropylidenediphenol esterification, which contain aromatic chains.

As observed in the fractographic analysis, the structure of the printed polymer is composed of layers, unlike the thermally activated acrylic, which contains inherent porosities from its manufacturing process,^38^These porosities impact the performance of the material in terms of water sorption. In thermally activated acrylic, the porosities concentrate stress and are occupied by water molecules following sorption, which alter the plasticity of the material.^27^ Conversely, the same effects may not occur in printed materials due to their layered polymeric network structures.^19^

Consistent with previous studies,^7,26,31^ the hardness of thermally activated acrylic was found to be superior to that of printed materials that were not subjected to double post-curing, and were not aged, partially rejecting the null hypothesis. This observation may indicate that the additive materials have a lower degree of conversion than the thermally activated resin.^7^ However, double post-curing benefited the additive groups (K), leading to an increase in the Knoop hardness, which aligned them with the conventional acrylic group (T), also leading to rejection of the null hypothesis. This may suggest that double post-curing improved the degree of conversion in the K group. Additionally, saliva can facilitate the enzymatic degradation of these polymers, with esterases promoting the esterification of methacrylates, subsequently affecting their hardness and resistance to wear.^4,30^ Artificial aging for 28 days did not influence the hardness of the materials, reflecting the same pattern observed in bending resistance. However, under 84 days of artificial saliva aging, the K additive material did not experience a significant decrease in hardness, in contrast to the other experimental and control groups, where aging negatively influenced their hardness, thereby rejecting the null hypothesis. In synergy with the observations regarding flexural strength, the water sorption primarily occurring in artificial saliva plasticizes the chains of the polymeric network, reducing hardness.^30^ The lower susceptibility of the K group to artificial aging may be attributed to differences in its polymeric network, which appears to be less prone to water sorption.^23-25,27, 30^

A total of 60,000 in vitro masticatory cycles is equivalent to approximately three months of in vivo use of occlusal splints.^3, 5^ The two-body wear assessment performed in this study simulated direct contact between the antagonist and the occlusal splint surface.^6^ In the literature,^4-7,9^ the load applied during the masticatory simulation ranges from 5 N to 50 N. Since other authors found no significant difference in wear between loads of 20 N and 50 N,^7^ a load of 20 N loads was selected for this study. Metallic spheres can serve as replacements for dental enamel antagonists,^1,4^ since they do not cause statistically significant differences in the wear promoted on the surface of the tested materials.^4^ One advantage of using resin,^4^ ceramic^6^ or dental enamel materials^3,5-7,9^ as antagonists is the ability to evaluate the wear to additive materials, which also occurs in the antagonist; this could be investigated in future studies. When comparing the K additive material to thermally activated acrylic resin, the K additive material demonstrated greater resistance to surface wear, thus rejecting the null hypothesis. This finding is contrary to a previous study,^2^ but corroborated in another.^4^ Although both referenced studies measured vertical losses, the discrepancies may be attributed to different parameters adopted in each study, such as the number of cycles (5,000–200,000) and load (5–15 N) used in shaping and polishing.^8,9^

It is expected that polymeric materials with greater hardness tend to exhibit greater wear resistance. ^7^ However, this study found that conventional acrylic material displayed greater surface microhardness than the additive materials, while experiencing similar or even less wear compared with the additive materials. This discrepancy may be attributed to the methods used to create the specimens, where differences in the internal structure — such as porosities and intrinsic defects — can affect thermally activated acrylic resin, potentially reducing its resistance to wear in deeper regions compared to its surface. In contrast, printed materials appear to exhibit greater homogeneity in their chains, both at the surface and internally, as evidenced in fractographic analysis.^4^ Similarly, while double post-polymerization increased the hardness of the K additive material, it did not optimize the wear resistance of any additive material. This observation may suggest that the post-curing of residual monomers occurs more intensely at the surface. This argument is further supported by the notion that when a solid is printed with very thick layers, the penetrability of UV light within the material may decrease, thus altering the absorption, and consequently reducing light-mediated post-polymerization within its interior.^19,24^

Although artificial aging for 84 days decreased the hardness of most of the tested materials (except the K84 group), the wear profile of these materials remained unaffected by artificial aging. This lack of impact suggests that water did not alter the structural loss caused by wear. These differences may be attributed to water sorption, plasticization, and enzymatic degradation occurring solely in the surface polymeric chains of the tested materials.^30^

Considering that uneven photopolymerization can lead to contractions resulting from the proximity of the monomers during the polymerization reaction,^20^the potential volumetric contraction occurring during the post-polymerization of the additive materials raises concerns regarding adaptation and stability. This instability may affect the frictional contact of the occlusal plate and even the dissipation of occlusal loads to the periodontium.^13,33-35^ Previous studies have shown that conventional post-curing did not influence dimensional accuracy,^22^ which corroborates the findings of this study. Although previous research^20^ indicates that variations in the duration or intensity of UV light in post-curing can cause distortions in the final solid, significant dimensional changes were not observed after doubled post-curing cycles either. These findings may indicate that, after printing, the desired degree of conversion is achieved with reduced polymerization shrinkage stresses, likely due to the composition and formation of the pre-designed polymeric network in the polymerization kinetics of these materials. ^19, 20^ While water sorption is a critical factor affecting the dimensional stability of the additive technology, the dimensional changes resulting from artificial aging were not statistically significant, indicating that these materials maintain dimensional stability even under conditions of water sorption.

## Conclusions

Based on the findings, limitations, and materials tested in this in vitro study, we can conclude that:

- The resistance to bending and dimensional change of additive materials is comparable to that of thermally activated materials.
- The microhardness of the additive materials is lower than that of the thermally activated materials.
- The patterns of wear and resistance to aging of the evaluated materials are similar, with the Kulzer additive demonstrating greater resistance to wear than the thermally activated material.
- The effectiveness of dual post-curing is dependent on the 3D printing system and material used; it can enhance the aging resistance of printed materials. Further studies are needed to investigate the conversion degrees and impact of different post-curing parameters.
- Doubling the post-polymerization time did not significantly change the conversion degree of the printed resins, and no statistical differences were observed between the conversion degree of the thermally activated acrylic resin and the printed resins.
